# Impact of phage therapy in post-weaning piglets challenged with ETEC strain in a controlled minitrial

**DOI:** 10.1186/s40813-026-00516-2

**Published:** 2026-04-29

**Authors:** Md. Rayhan Mahmud, Md. Karim Uddin, Peppi Kareljärvi, Matti Jalasvuori, Juuli Peräkylä, Tiia Eklund, Mia Biström, Shah Hasan, Tommi Vatanen, Saija Kiljunen, Claudio Oliviero

**Affiliations:** 1 Department of Production Animal Medicine, Faculty of Veterinary Medicine, University of Helsinki, Helsinki, Finland; 2 Institute of Biotechnology, Helsinki Institute of Life Science, University of Helsinki, Helsinki, Finland; 3 Department of Microbiology, Faculty of Agriculture and Forestry, University of Helsinki, Helsinki, Finland; 4 Broad Institute of MIT and Harvard, Cambridge, MA USA; 5 Liggins Institute, University of Auckland, Auckland, New Zealand; 6 Department of Biological and Environmental Science, Nanoscience Center, University of Jyväskylä, Jyväskylä, Finland; 7 Human Microbiome Research Program, Research Programs Unit, Faculty of Medicine, University of Helsinki, Helsinki, Finland; 8 European Society of Clinical Microbiology and Infectious Diseases (ESCMID) Study Group for Non-Traditional Antibacterial Therapy (ESGNTA), Basel, Switzerland; 9 Finnish Food Authority, Animal Health Diagnostic Unit, Helsinki, Fl-00790 Finland

**Keywords:** Bacteriophage, *Escherichia coli*, Gut microbiota, Post-weaning diarrhea (PWD), Zinc oxide (ZnO), Phage therapy

## Abstract

*Enterotoxigenic Escherichia coli* (ETEC) is a pathogen responsible for post-weaning diarrhea (PWD) in piglets, which results in economic losses in pig production. The rise of antibiotic-resistant ETEC strains together with restrictions on addition of zinc oxide in pig feed require alternative management approaches. Our research examines bacteriophage therapy as a solution to control ETEC infections in newly weaned piglets. A cocktail of phages targeting a strain of ETEC F4LT1ST2 was identified and subsequently multiplicated in laboratory. We conducted a trial including nine piglets divided into three groups. The negative control group was exposed to the phage cocktail by administration with the bedding material (saw dust) on the floor of their pen. The treatment group was exposed to the ETEC strain and to the phage cocktail, and the positive control group was exposed to the ETEC strain only. Shotgun metagenomic sequencing was performed on fecal samples to characterize bacterial and phage dynamics. Throughout a 10-day period we monitored daily the rectal temperature and the diarrheal score of piglets. Subsequently we evaluated phage and bacterial counts in fecal samples to determine phage therapy effect on gut microbiota dynamics and piglet health. The PHAGE+ETEC group showed 19.2% lower cumulative diarrhea burden (*p* = 0.044) and 61.9% higher average daily gain (*p* = 0.065). Rectal temperature correlated significantly with diarrhea severity (per-piglet Spearman’s ρ = 0.727, *p* = 0.027). Alpha diversity did not differ between treatment groups across timepoints, suggesting that phage administration did not cause major shifts in microbial diversity. Metagenomic analyses showed significant reduction of *E. coli* abundance in PHAGE+ETEC group compared to PHAGE groups (*p* = 0.009). Consistent with these observations, plaque assay results confirmed active phage-bacteria interactions: no plaque formation was detected in the feces of the ETEC-only group, whereas the PHAGE+ETEC group showed phage replication, reaching 10^6^ PFU/ml. This pilot study highlights the potential of phage therapy as an alternative to antibiotics for ETEC infections in piglets. Additional research with larger pig population and longer duration is required to confirm these findings and develop optimal phage application methods for swine production.

**Supplementary information:**

The online version contains supplementary material available at 10.1186/s40813-026-00516-2.

## Introduction

ETEC (Enterotoxigenic *Escherichia coli*) stands out as a major cause of diarrheal disease in humans and various animal species [1]. Post-weaning diarrhea (PWD), a life-threatening disease (takes place during the first 2 weeks of weaning) in piglets is caused by ETEC and results in increased use of antimicrobials and in substantial economic losses in global pig production. These losses result from increased mortality rates, reduced growth performance, and elevated veterinary costs. ETEC strains are well-known to generate enterotoxins including heat-stable (ST) and heat-labile (LT) toxins that seriously damage intestinal function leading to watery diarrhea [2, 3]. The genetic diversity and various virulence factors of ETEC strains complicate efforts to prevent and control infections, making it a persistent challenge for pig farms, especially those employing intensive farming practices [1, 4].

Intestinal infections in young and post-weaned piglets have traditionally been counteracted improving management practices before and at weaning, and during outbreaks through antibiotic use [5]. The widespread use of antibiotics has resulted in the development of antibiotic-resistant ETEC strains that threaten both animal and human health [6]. The European Union (EU) has established rigorous rules to decrease antibiotic usage in livestock because of this concern [7]. In addition, in ETEC chronically affected farms and to control PWD and to enhance the growth performance of piglets, zinc oxide (ZnO) has been widely used in feed. However, since June 2022, the EU has restricted the use of ZnO throughout the Europe for its adverse environmental impact and developing antimicrobial resistance microorganisms resulting from high dose usage [8, 9]. The regulatory change emphasizes the need to establish environmentally friendly solutions for PWD management.

Bacteriophage therapy has emerged as an alternative treatment because it targets pathogenic bacteria effectively without harming beneficial microbiota [10]. Bacteriophages are viruses that specifically attack bacteria and demonstrate potential as antibiotic replacements. The precise nature of their target mechanism combined with their ability to evolve along with bacterial hosts decreases the probability of resistance formation [11]). Bacteriophages can be obtained from natural environments and engineered to focus on particular bacterial strains which improves their application for bacterial infection treatment [10, 11]. A recent mouse studies showed that intragastric inoculation of phage into 12 hours post inoculated ETEC mice reduced gastrointestinal disorder and mortality rate. Other studies showed that treatments with lytic bacteriophages have been able to reduce *E. coli, Salmonella, and Campylobacter* in infected poultry and to reduce animal mortality [12].

Bacteriophage therapy seems a promising alternative to antibiotics, yet researchers have not fully explored its use in pig production systems. Although recent research indicates its effectiveness in controlling bacterial diseases in livestock populations, there is still a lack of extensive studies about its specific impact on ETEC infections in piglets [12, 13]. Therefore, the main goal of this study was to isolate bacteriophages effective against diffuse ETEC strains that are found in Finnish pig farms and to use these phages for the treatment of ETEC -induced diarrhea. We also aimed to verify if phages active against a specific ETEC strain, would be effective if administered preventively in the bedding material given to the piglets exposed to that specific ETEC strain.

We hypothesized that the phages would reduce ETEC-related symptoms and diarrhea incidence.

The long-term aim of the phage therapy is to minimize disease symptoms, mortality and antibiotic prescription in pig farming.

The final aim was to determine if phage therapy would have any evident effect on the gut microbiome. Achieving these objectives will create a sustainable antibiotic alternative which will improve both animal health and farm productivity.

## Methodology

### Bacterial strains, culture media, and basic microbiological methods

Enterotoxigenic *E. coli* strain (ETEC F4LT1ST2) was obtained from the collection of Finnish Food Authority. Bacterial cultures were carried out at 37 °C in Luria-Bertani (LB) medium and supplemented with 1.5% (w/v) or 0.7% agar for LB-agar and soft-agar media, respectively [14]. To culture bacteria for animal experiments, one colony of ETEC F4LT1ST2 was inoculated into 20 ml of LB and cultured at 180 rpm for five to six hours. Of this preculture, 1 ml was transferred to each of four 500-ml aliquots, which were cultured overnight and used in the animal experiment the same day. The concentration of viable bacteria in the cultures was determined by plating 100-µl aliquots of serial dilutions of the cultures onto LB-agar plates and calculating the colonies after an overnight incubation.

### Isolation, characterization, and production of phages used in the work

Pig fecal samples collected earlier from Finnish pig farms were used as the starting material for bacteriophage isolation. Fecal suspensions were prepared in LB medium and clarified by filtration through 0.5 µm pore-size filters to remove bacterial cells while retaining viral particles. The host bacterium *E. coli* strain F4LT1ST2 was cultivated in LB medium under standard conditions (37 °C shaken at 200 rpm). For enrichment, 200 µL of the host culture was added to the filtered fecal suspension, followed by the addition of 5 mL of LB medium. The enrichment mixtures were incubated overnight to allow phage amplification. Following enrichment, aliquots of the cultures were sampled and spotted or plated on lawns of ETEC F4LT1ST2 to assess the presence of lytic phages by plaque formation. Individual plaques were picked and replated on the strain three times in order to ensure that a single phage was selected. Following phages were selected for use in animal experiment: fPigEC39p8, fPigEC39p10, fPigEC39p13, fPigEC39p20, fPigEC39p21.

Phage production for the pig experiment was performed as follows: *E. coli* strain F4LT1ST2 was cultivated in 5 ml of LB. 800 microliters of overnight culture was transferred to 800 ml of fresh LB and cultivated in 37 °C under shaking (110 rpm) in 2 litre erlenmayer flasks. After two hours of cultivation, 4 ml of each of the phage stocks fPigEC39p8 (1.9 × 10^9^ PFU/ml), fPigEC39p10 (3.9 × 10^9^ PFU/ml), fPigEC39p13 (7.0 × 10^8^ PFU/ml), fPigEC39p20 (5.0 × 10^8^ PFU/ml), fPigEC39p21 (3.0 × 10^8^ PFU/ml) was added individually to the 800 ml bacterial culture. After 16 hours cultivation under shaking 100 rpm, bacteria were removed from the phage-bacteria co-cultures by first centrifugation for 25 minutes with 6000 rpm and then filtering through Nalgene rapid flow 0.45 micrometer filters. All phage productions were mixed together in three separate mixtures, each containing all the phages in equal volumes. 450 ml of the phage mixture was transferred in sterile conditions to 500 ml bottles and stored in + 7 °C for subsequent use in animal experiment. Total of 20 litres of phage mixture was prepared, with the titer 1.2 × 10^9^ PFU/ml.

### Experimental design

**Fig. 1:**
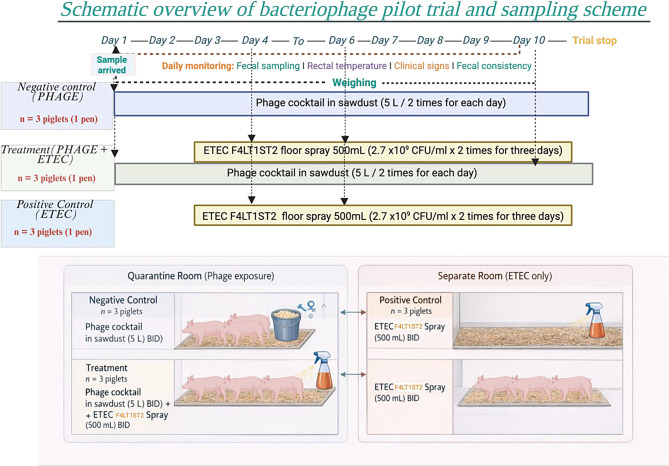
Experimental design and sampling scheme of the bacteriophage pilot trial (created in [15])

The experimental timeline, group allocation, housing arrangement, and treatment procedures are summarized in Fig. 1.This study was performed at Production Animal Veterinary Hospital in Saari Finland (Faculty of Veterinary Medicine, University of Helsinki). Nine healthy 4-week-old piglets were collected from a commercial farm near the experimental location. At the arrival to the experimental facilities, the piglets were allocated to three different pens: 3 ×negative control = exposed to phages only, 3 ×treatment = exposed to phages + ETEC F4LT1ST2, and 3 ×positive control = exposed to ETEC F4LT1ST2 only. The positive control group was kept in a pen for sick animals of the hospital, while the two other piglet groups (negative control and treatment) were housed in a completely isolated quarantine room to avoid accidental phage contamination of the positive control piglets. Negative control and treatment groups got phage cocktail mixed in 5 liters of sawdust twice a day for ten days. Positive control and treatment groups were infected with ETEC F4LT1ST2 by spraying 500 ml culture (2.7 × 10^9^ CFU/ml) on the floor of the corresponding pens uniformly twice a day for three consecutive days (days 3 to 5) (Fig. 1).

### Monitoring fecal samples and piglet wellbeing

Piglets were sampled daily for feces, rectal temperature (was taken as first when manipulating the piglets), and monitored for clinical signs and feces consistency (visual score and dry matter measurement). Fecal samples were collected using cotton swabs inserted into the rectum and placed into sealable plastic bag (Minigrip, USA). All samples were immediately frozen on dry ice for transportation to the laboratory and stored at −70 °C until further processing. A digital human scale (KROKEK, Sweden) was used for weighing of the piglets during first and last days of the experiment.

### Growth performance measurement

Piglets were weighed at the beginning (day 0) and end (day 10) of the experimental period using a calibrated digital scale (KROKEK, Sweden). Average daily gain (ADG) was calculated as: ADG (g/day) = (Final weight - Initial weight)/Number of days.

### Fecal ETEC cultivation and PCR

Fecal samples were transported to diagnostic laboratory the same day of sampling. The fecal samples were streaked into ovine blood agar plates and incubated aerobically in 37 °C. After 16–24 hours of incubation, plates were examined for the presence of *E. coli* bacteria, and the amount and purity of *E. coli* growth were assessed visually. Bacterial species identification was carried out by MALDI-TOF MS (matrix assisted laser desorption ionization–time of flight mass spectrometry, Bruker Biotyper®, Bruker Daltonics GmbH, Germany with MBT Compass Library updates 2020). *E. coli* isolates were tested with PCR for fimbrial (F4, F6, F18) and toxin genes (LT1, ST1, ST2, Stx2e) Wieler and Bauerfeind [16].

### Presence of phages in fecal samples

Presence of phages in fecal samples was investigated by transferring approx. 1 ml of fecal matter to 3 ml of sterile water, mixed well by vortexing and filtered with 0.5 micrometer filter to remove bacteria. 200 µl of this water-feces mixture was mixed with 200 µl of overnight grown ETEC F4LT1ST2 and plated in 0.7% agar on LB-agar plates. Formed plaques were counted after overnight culture.

### Microbial DNA extraction and sequencing

Bacteriophage DNA was isolated from crude phage lysate with Phage DNA Isolation Kit (Norgen Biotek) and ETEC genomic DNA was isolated from 1 ml of overnight culture using JetFlex™ Genomic DNA Purification Kit. DNA concentration was measured with QubitTM Fluorometer 4.0 and QubitTM Broad Range DNA assay kit (Invitrogen by Thermo Fisher Scientific, USA). The sSequencing of both phage and bacterial genomes was performed with Illumina platform using NovaSeq X Plus Series (PE150) at Novogene Europe.

For metagenomic analyses, microbial genomic DNA was extracted using DNeasy PowerSoil Pro Kit (Qiagen, cat. no. 47014, Hilden, Germany) from 125 mg of piglet fecal material in accordance with the manufacturer’s instructions (Qiagen, Hilden, Germany). An extraction blank, which is an empty sampling device subjected to the complete DNA extraction protocol without any biological input, was processed concurrently with samples to identify potential contamination from reagents or laboratory handling. A Nanodrop 2000 spectrophotometer (Thermo Fisher Scientific, Waltham, MA, USA) was used to measure the concentration and purity of the extracted DNA. To perform whole metagenome sequencing of the extracted microbial DNA, 100 ng of genomic DNA was converted to sequencing library using the Illumina DNA prep. Samples were dual indexed using IDT for Illumina UD Indexes (Illumina). In the protocol, 7 cycles were used in the PCR step and DNA was pooled and purified using Illumina’s SPB bead purification. The library pool was sequenced at 9 pM on the AVITI sequencer (Element Biosciences) using the AVITI 2 × 150 Sequencing kit Cloudbreak FreeStyle High Output.

### Bioinformatics analyses

Bacteriophage isolate genomes were assembled, annotated and analyzed in Phagenomics -portal version 1.0 (http://www.phagenomics.net). For each genome, Phagenomics assembled the genome from raw Illumina reads with SPAdes genome assembler v3.15.5. The system selected the biggest contig with the highest coverage and confirmed that the contig is of viral origin. Annotation utilized the Phagenomics annotation pipeline that employs several tools to identify putative functions for open reading frames, namely PhANNs and Prokka version 1.14.6. The predicted open reading frames were annotated against Phrogs, COGs, pVOGS, and Uniprot databases, and consensus annotations were generated by Phagenomics. To predict ETEC serotypes of the individual isolates, raw reads were submitted to, and the analysis was carried out at EnteroBase [17].

Paired-end metagenomic sequencing data underwent a comprehensive quality control and host decontamination using Trim Galore v0.6.10 (ILLUMINACLIP (2:30:10:1:true), SLIDINGWINDOW (4:15), HEADCROP (15), CROP (135), MINLEN:50), and KneadData v0.12.0 (default settings). Taxonomic profiling was performed using MetaPhlAn4. Modular Viromics Pipeline v.1.1.4 was used pipeline for phageome analysis. The pipeline executed viral identification and quality assessment through geNomad and CheckV, followed by ANI clustering at 95% sequence identity generating viral Operational Taxonomic Units (vOTUs).

### Statistical analyses

Non-parametric methods were primarily employed due to the inherently non-normal distribution of both bacterial and phage abundances.

Non-parametric tests were used throughout due to small sample size (*n* = 3/group). Kruskal-Wallis tests for group comparisons in case of clinical outcomes, Spearman’s rank correlation for temperature-diarrhea association, and Cohen’s d effect sizes for pairwise treatment comparisons. Microbiome and phageome: alpha diversity (Shannon index and vOTU richness) was compared using Kruskal-Wallis tests both overall and per timepoint, with Dunn’s test for pairwise post-hoc comparisons using Benjamini-Hochberg (BH) correction for multiple testing. Beta diversity was assessed using Bray-Curtis dissimilarity matrices (vegan package v2.6–4 in R v4.2.0), visualized by principal coordinate analysis (PCoA), and tested using PERMANOVA (adonis2) with a full factorial model (Treatment × Day). To account for the repeated-measures design (each piglet sampled at three timepoints), permutations were restricted within piglets using the strata argument (permute package), ensuring that the non-independence of samples from the same animal was properly handled (999 restricted permutations). Homogeneity of multivariate dispersions was verified using betadisper with permutation tests (999 permutations) to confirm that PERMANOVA results reflected true location differences rather than dispersion artifacts. Pairwise PERMANOVA was conducted per timepoint to assess treatment effects at individual sampling days. *E. coli* abundance was compared using Kruskal-Wallis followed by Dunn’s post-hoc test (BH-adjusted); pairwise Mann-Whitney U tests with BH correction were also performed for effect size reporting. Phageome RPKM values were converted to TPM for normalized abundance comparisons. Significance threshold: α = 0.05; trends noted at *p* < 0.10. All analyses were performed in R v4.2.0.

## Results

### Phages used in the work

Despite several attempts to isolate phages against the ETEC F4LT1ST2 strain, all phages that we were able to obtain were similar to phages in genus *Lederbergvirus.* These are temperate phages having approx. 40–41 kb genomes, most famous member of this group being P22 [18]. However, as phages fPigEC39p8, fPigEC39p10, fPigEC39p13, fPigEC39p20, and fPigEC39p21 formed clear plaques, grew to high titers in the ETEC F4LT1ST2 strain, and their genomes did not contain genes involved in bacterial virulence or antibiotic resistance, we reasoned that they can be used in this closed phage therapy trial.

### Diarrhea, temperature, growth performance, and fecal ETEC profiles in piglets following bacteriophage treatment

**Fig. 2:**
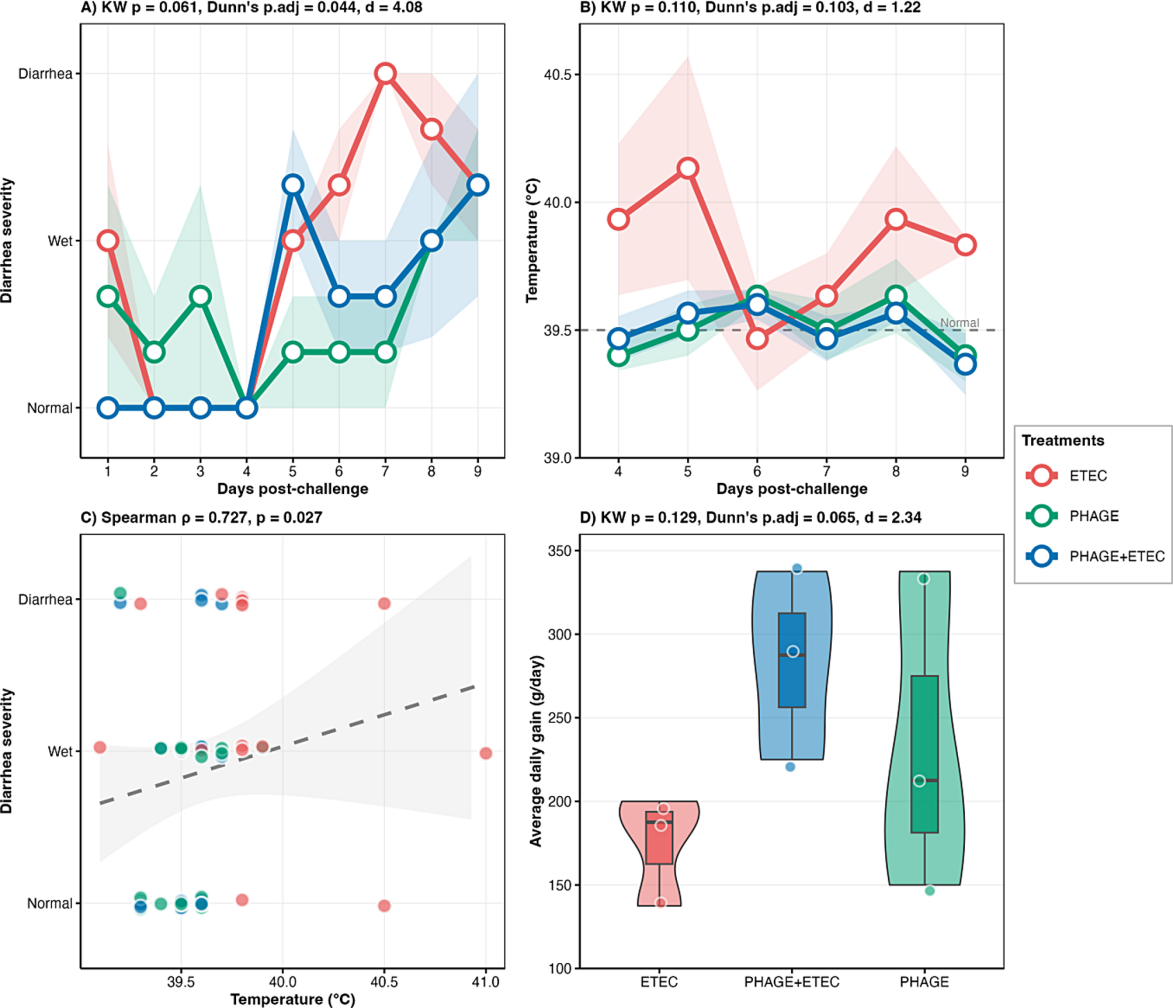
Clinical outcomes of phage therapy in ETEC-challenged piglets. (**A**) Diarrhea severity progression over 9 days post-challenge, showing reduced burden in PHAGE+ETEC group (Kruskal-Wallis *p* = 0.061, Cohen’s d = 4.08). (**B**) Rectal temperature (Days 4–9) remained closer to normal (dashed line) in phage-treated animals (*p* = 0.110, d = 1.22). (**C**) Significant correlation between temperature and diarrhea severity validates temperature as a disease biomarker (Spearman’s ρ = 0.304, *p* = 0.025). (**D**) Average daily gain demonstrated substantial improvement with phage therapy (*p* = 0.129, d = 2.34). Data shown as mean ± SEM

Prophylactic phage administration significantly impacted multiple clinical outcomes in ETEC-challenged piglets. PHAGE+ETEC animals demonstrated 19.2% lower cumulative diarrhea burden compared to ETEC controls, (Kruskal-Wallis *p* = 0.061). Dunn’s post-hoc test with Benjamini-Hochberg correction confirmed a significant pairwise difference between ETEC and PHAGE+ETEC (p.adj = 0.044, Cohen’s d = 4.08). ETEC vs. PHAGE was also significant (p.adj = 0.045, d = 1.74), while PHAGE+ETEC vs. PHAGE did not differ (p.adj = 0.382)(Fig. 2A). The temporal pattern revealed that diarrhea severity peaked at Day 7 post-challenge in ETEC-infected animals (mean severity = 3.0, watery diarrhea), while PHAGE+ETEC piglets maintained consistently lower severity throughout the monitoring period. PHAGE-only controls showed minimal diarrhea, confirming effective ETEC challenge in treatment groups.

Mean rectal temperature was 0.32 °C lower in PHAGE+ETEC versus ETEC groups across the Day 4–9 measurement period (Kruskal-Wallis *p* = 0.110, d = 1.22) (Fig. 2B), indicating reduced systemic inflammatory response. Dunn’s post-hoc: ETEC vs. PHAGE+ETEC p.adj = 0.103 (trending); ETEC vs. PHAGE p.adj = 0.052 (trending).

Importantly, temperature significantly correlated with diarrhea severity across all treatment groups (Spearman’s ρ = 0.727, *p* = 0.025) (Fig. 2C), validating rectal temperature as a non-invasive biomarker for enteric disease severity.

Growth performance showed marked improvement with phage therapy. Average daily gain tend to increase 61.9% in PHAGE+ETEC piglets (285.7 ± 62.3 g/day) compared to ETEC controls (177.4 ± 22.0 g/day) (Kruskal-Wallis *p* = 0.129; Dunn’s p.adj = 0.065, d = 2.34) (Fig. 2D). This translates to 1.08 kg additional body weight over the 10-day experimental period, representing substantial economic benefit in commercial swine production.

We observed the presence of targeted ETEC strain (F4LT1ST2) in one piglet in the ETEC challenged group by the fecal culture and subsequent PCR confirmation. Along with the targeted profile, other ETEC virulence profile (F18ST1ST2) was detected in four piglets (two in ETEC group, one in Treatment group, and one in Phage group) (Table 1). The complete strain-level PCR results are provided in Supplementary Table S1.This finding is likely attributable to background or environmental exposure rather than experimental infection.

**Table 1:** Fecal culture results (ETEC profiles) and diarrhea outcomes

Piglet	Group	Bacteriological diagnosis	Comment	Diarrhea
1	ETEC	Escherichia coli (ST1, ST2, F18)	abundant and almost pure	Yes
2	ETEC	Escherichia coli (ST1, ST2, F18)	abundant with mixed growth	Yes
3	ETEC	Escherichia coli (LT1, ST2, F4)	abundant and almost pure	Yes
4	PHAGE+ETEC	Escherichia coli (ST1, ST2, F18)	abundant with mixed growth	Yes
5	PHAGE+ETEC	Specific infection not detected.	only a few hemolytic colonies	No
6	PHAGE+ETEC	Specific infection not detected.	only a few hemolytic colonies	No
7	PHAGE	Specific infection not detected.	only a few hemolytic colonies	No
8	PHAGE	Escherichia coli (ST1, ST2, F18)	abundant and almost pure	Yes
9	PHAGE	Specific infection not detected.	no hemolytic colonies	No

The serotypes of both targeted F4LT1ST2 and contaminating F18ST1ST2 ETEC strains were predicted in EnteroBase. For F4LT1ST2 strain, the prediction was O149:H10, whereas the F18ST1ST2 isolates were predicted to be O149:H40. Even though all strains had O149 serotype, their phage susceptibility was different. The targeted F4LT1ST2 strain was infected clearly by all phages used in the phage cocktail, but F4LT1ST2 isolates were resistant to all but one phage. Furthermore, even though one of the F18ST1ST2 isolates was infected by phage fPigEC39p8, the infection was weak.

### Bacteriophage plaque formation in piglet fecal samples

**Fig. 3:**
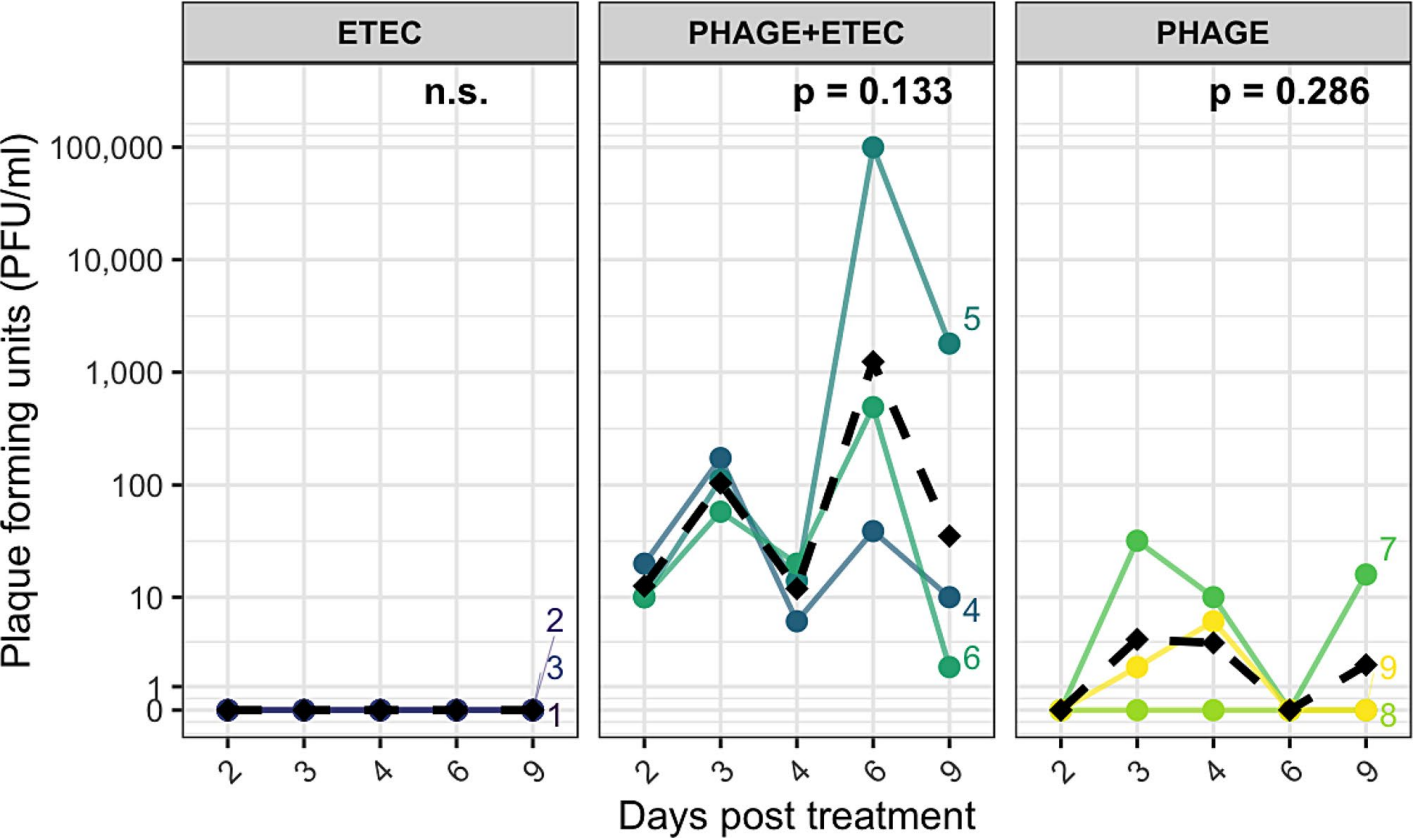
Longitudinal observation of bacteriophage plaques formation in piglet feces by whole plate test. We looked at three treatment groups over five days: ETEC-only (positive control), PHAGE+ETEC (treatment group), and PHAGE-only (control for phage persistence). Colored lines show the paths of each piglet, and dashed black lines represent group means of the piglets

The longitudinal graph shows that the three treatment groups (ETEC, Treatment, and Phage) had distinct patterns of bacteriophage plaque formation over time (Fig. 3). We didn’t observe any visual appearance of bacteriophage plaque formation (0 PFU/ml) in ETEC treated piglets at any time points. However, we visualized substantial phage replication in the PHAGE+ETEC groups with high variation between individuals, specifically evident in piglet 5 reaching about 10^6^ PFU/ml. The Phage-only group showed low but detectable levels, with values between 0 and 32 PFU/ml. Interestingly, piglet 8 having the contaminant F18ST1ST2 isolate had 0 PFU/ml at all time points, giving further support to the finding that this strain was not infected by the phages used in the work. The Kruskal-Wallis tests didn’t find any significant temporal differences within groups (Treatment: *p* = 0.133; Phage: *p* = 0.286). This is probably because the sample size was too small and there was too much biological variability. The key finding is that the Treatment group had a higher plaque formation than either of the control groups. This indicates that the phage was able to replicate successfully in the presence of the bacterial host (ETEC F4LT1ST2 strain).

### Microbiome diversity and composition

**Fig. 4:**
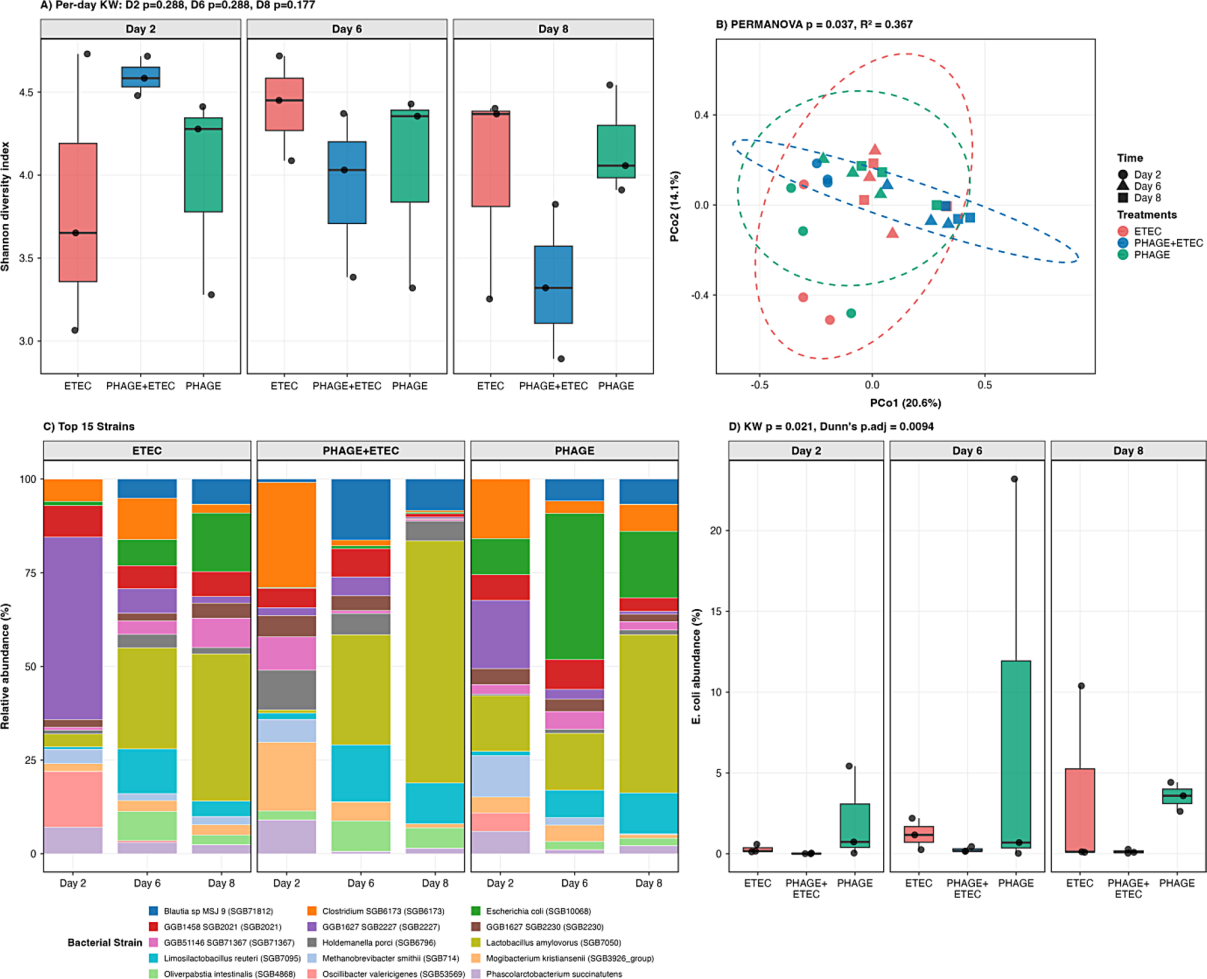
Bacterial community diversity and composition across treatment groups. (**A**) Shannon diversity index treatment groups (ETEC, PHAGE+ETEC, and PHAGE) on days 2, 6, and 8. Box plots show median, interquartile range, and outliers. (**B**) Principal coordinate analysis (PCoA) of Bray-Curtis dissimilarities showing the relationships between bacterial communities across treatments and time points. (**C**) Relative abundance of top 15 taxa across treatment groups and time points. (**D**) Boxplots showing the relative abundance of E. coli strain (cladeSGB10068) across treatment groups on days 2, 6, and 8

Bacterial alpha diversities, measured by Shannon index, did not significantly differ between treatment groups at any timepoint (day 2: *p* = 0.29; day 6: *p* = 0.29; day 8: *p* = 0.18) (Fig. 4A).

Bray-Curtis dissimilarity revealed partial treatment-specific clustering (PCo1: 20.6%, PCo2: 14.1%). PERMANOVA with strata-restricted permutations (accounting for repeated sampling of piglets) detected a significant Treatment × Day interaction (F = 1.303, R^2^ = 0.367, *p* = 0.037), though individual timepoints were non-significant (Day 2: *p* = 0.258; Day 6: *p* = 0.403; Day 8: *p* = 0.306). Betadisper confirmed homogeneity of multivariate dispersions for both Treatment (F = 0.055, *p* = 0.933) and Day (F = 0.368, *p* = 0.685), validating the PERMANOVA results as reflecting true compositional differences (Fig. 4B).

The 15 most abundant taxa shifted over time and between treatments (Fig. 4C). *E. coli* (SGB10068) was present in all samples, with the PHAGE group showing the highest abundance at day 6.

E. coli abundences were significantly different among treatments (Kruskal-Wallis χ^2^ = 7.728, *p* = 0.021). PHAGE+ETEC had the lowest E. coli (0.137%) vs. ETEC (1.677%) and PHAGE (4.526%). Dunn’s post-hoc test (BH-adjusted): PHAGE+ETEC vs. PHAGE (p.adj = 0.009); ETEC vs. PHAGE+ETEC (p.adj = 0.053), Mann-Whitney U with BH correction corroborated these findings (PHAGE+ETEC vs. PHAGE: p.adj = 0.032; ETEC vs. PHAGE+ETEC: p.adj = 0.076) (Fig. 4D).

### Phageome diversity and composition

**Fig. 5:**
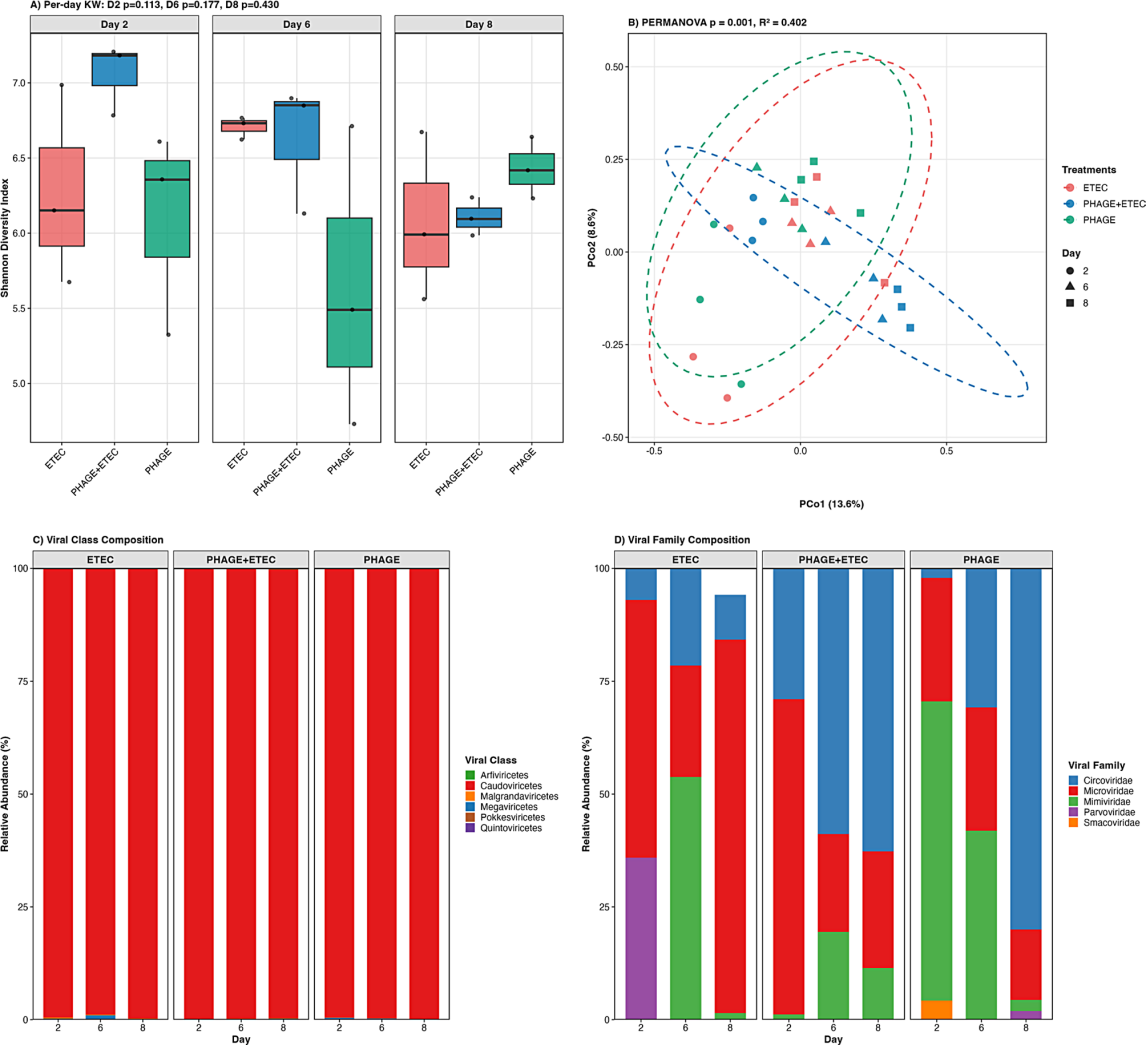
Phage community diversity and composition across treatment groups. (**A**) Shannon diversity index for phage communities across treatment groups (ETEC, PHAGE+ETEC, and PHAGE) on days 2, 6, and 8. (**B**) Principal coordinate analysis (PCoA) of Bray-Curtis dissimilarities showing clustering of phage communities by treatment group. (**C**) Class-level composition of phage communities across treatment groups and time points, showing the predominance of Caudoviricetes. (**D**) Family-level composition of phage communities showing temporal shifts in phage taxonomy across treatment groups

Phageome diversity, measured by Shannon index, ranged from 4.73 to 7.21. The PHAGE+ETEC group had the highest alpha diversity at day 2 (mean = 7.0), which declined by day 8. PHAGE diversity dipped at day 6, then recovered. Despite these observed trends, Kruskal-Wallis tests revealed no statistically significant differences between treatment groups at any time point (day 2: *p* = 0.113; day 6: *p* = 0.177; day 8: *p* = 0.430). (Fig. 5A).

In Beta diversity, PCoA ordination captured 22.2% of the total variance (PCo1: 13.6%, PCo2: 8.6%), with apparent differences by treatment group (Fig. 5B). PERMANOVA test in beta-diversities (Bray-Curtis dissimilarity) revealed significant differences in phage community structure between treatment groups (Treatment × Day interaction; *p* = 0.001, F = 1.511, R^2^ = 0.402)(Fig. 5B).

Caudoviricetes was the dominant phage class with relative abundance over 95% across all samples (Fig. 5C). On family level, Microviridae dominated in PHAGE+ETEC on days 2 and 8, Circoviridae was prevalent in ETEC by days 6 and 8, and PHAGES had higher levels of Mimiviridae and Smacoviridae at day 2 (Fig. 5D). Temporal shifts in community composition appeared to correspond to bacterial colonization and phage intervention timing.

## Discussion

In this study, we demonstrated the impact of phage therapy for treating ETEC-induced diarrhea in post-weaning piglets and determining its effects on the gut microbiome and piglet health. The pilot study utilized a floor-based challenge and treatment model, contrasting with prior research that primarily employed oral gavage, to better reflect natural transmission dynamics observed in farm environments. Although the limited sample size constrains statistical power; however, the study offers integrated clinical, microbiological, and multi-omics observations that collectively inform future trial design. Key findings of our study: (i) ETEC-specific phage treatment in piglets resulted in decreased diarrhea occurrences and improved piglet growth which demonstrates phage therapy as a promising therapeutic approach for ETEC infections. (ii) Phages in a liquid solution can be administered to piglets by mixing it with the bedding material like saw dust, allowing a practical, affordable, and effective use in commercial pig farms. (iii) Phage treatment did not alter alpha diversity of gut microbiota which indicates that the native microbial community remained intact. (iv) phage community profiles between groups showed distinct compositions where Caudoviricetes emerged as the dominant phage group which demonstrated intricate phage-host relationships.

We observed 19.2% diarrhea reduction (Cohen’s d = 4.08, *p* = 0.061), which aligns closely with phage therapy efficacy in ETEC-challenged pigs. Cha et al. [19] demonstrated that phage-treated pigs showed enhanced diarrhea resistance on day 3 post-challenge with 60–64% bacterial reduction, paralleling our clinical improvements. Similarly, Jamalludeen et al. [20] reported significant reductions in diarrhea duration and severity scores following prophylactic and therapeutic phage administration, supporting our findings of large effect sizes despite modest sample size. Our 61.9% growth improvement (d = 2.34) exceeds typical outcomes, as recent studies report mixed results. While Li et al. [21] observed significant weight gain with phage cocktails reducing E. coli load by 1.04–1.17 log units, Imklin et al. [22] found no significant ADG differences despite 1.33 log-unit bacterial reduction. This variability likely reflects phage formulation differences, challenge timing, and gut microbiome modulation effects. The significant temperature-diarrhea correlation (ρ = 0.304, *p* = 0.025) validates rectal temperature as a non-invasive biomarker for ETEC disease severity.

This per-piglet analysis provides a conservative estimate that accounts for repeated-measures pseudoreplication inherent in daily observation data. This finding has practical implications for on-farm disease monitoring, as temperature measurement is simpler and less subjective than fecal scoring. To our knowledge, this is among the first studies to formally quantify this relationship in the context of phage therapy trials, providing a useful metric for future study designs.

Even though our study was complicated by the contaminating ETEC strain present in some of the piglets at their arrival, the overall study showed the potential of phage therapy to reduce ETEC infections in post-weaning piglets. Diarrhea incidence was found to be lower in the PHAGE+ETEC group than in the ETEC only group, although due to the low number of piglets used not statistically significant. This result aligns with previous research which have shown the effectiveness of phage therapy in decreasing bacterial infections in animal models. For example, Cha et al. observed that treating ETEC infected pigs with ETEC-specific phages less likely to get diarrhea [19]. Jamalludeen et al. also reported that a phage cocktail designed to target ETEC O149:H10: F4 could reduce the incidence of diarrhea in weaned piglets [20].

Bacterial alpha diversity, analyzed in this study did not show any significant differences between treatment groups, which means that phage therapy did not cause any disruption of the overall gut microbial community. These results are in agreement with previous studies which have shown that phages are pathogen-specific, leaving other microorganisms intact [23]. The treatment-specific clustering revealed by the beta diversity metrics indicates that phages changed the microbial community.

In terms of virome dynamics, we found substantial shifts in phage community structure among the groups. The *Caudoviricetes* phage class was the most prevalent; however, phage taxonomy shifts corresponded with bacterial colonization and temporal changes in phage types were observed. These observations indicate that phage-host interactions are dynamic and may respond to microbial fluctuations as recently shown in the field of gut virome ecology [24]. Although the administered phages were present in low numbers, their steady presence, particularly in the PHAGES-only group, suggests at least transient establishment. This is consistent with a recent finding which detected oral microencapsulated supplemented phages in the gut and associated with *E. coli* reduction in weaned piglets [25].

Despite positive trends, the limitations of a small sample size, short study period, and contaminating ETEC strain that was not sensitive to the phage cocktail, reduced our ability to achieve statistically significant results. Similar issues have been raised in previous studies on livestock phage therapy [26–28], which highlights the requirement for larger, longer-term studies to confirm results and examine the effects on growth, immunity, and productivity.

## Conclusion

In conclusion, our study highlights the potential of phage therapy as a targeted, sustainable, and safe alternative to antibiotics for controlling ETEC infections in piglets. By lowering disease symptoms and antibiotic consumption, phage therapy can improve animal health and sustainable pig farming, which leads to increased farm productivity. With the EU-wide ban on zinc oxide in pig feed (European Commission, 2021), urgency of immediate non-antibiotic interventions in pig production is growing [29]. The present small-scale trial establishes research potential while demonstrating phage therapy’s effectiveness in reducing antibiotic use in livestock farming and that bedding material (saw dust) can be used as a practical vehicle of phage therapy in pig farms. Future research requires larger well-powered studies to confirm these results and develop practical treatment protocols for pig production systems. [17, 30–42]

## Electronic supplementary material

Below is the link to the electronic supplementary material.

Supplementary Material 1

## Data Availability

The data sets used in this study are available in the European Nucleotide Archive (ENA) under accession PRJEB98680.
